# Bovine Pericardium Patch Wrapping Intestinal Anastomosis Improves Healing Process and Prevents Leakage in a Pig Model

**DOI:** 10.1371/journal.pone.0086627

**Published:** 2014-01-29

**Authors:** Mario Testini, Angela Gurrado, Piero Portincasa, Salvatore Scacco, Andrea Marzullo, Giuseppe Piccinni, Germana Lissidini, Luigi Greco, Maria Antonietta De Salvia, Leonilde Bonfrate, Lucantonio Debellis, Nicola Sardaro, Francesco Staffieri, Maria Rosaria Carratù, Antonio Crovace

**Affiliations:** 1 Department of Biomedical Sciences and Human Oncology, Unit of Endocrine, Digestive and Emergency Surgery, University Medical School “A. Moro”, Bari, Italy; 2 Department of Biomedical Sciences and Human Oncology, Unit of Medicine “A. Murri”, University Medical School “A. Moro”, Bari, Italy; 3 Department Basic Medical Sciences, University Medical School “A. Moro”, Bari, Italy; 4 Department of Emergency Surgery and Organ Transplantation, Unit of Pathology, University Medical School “A. Moro”, Bari, Italy; 5 Department of Emergency Surgery and Organ Transplantation, Unit of General Surgery and Liver Transplantation, University Medical School “A. Moro”, Bari, Italy; 6 Department of Biomedical Sciences and Human Oncology, Section of Pharmachology, University Medical School “A. Moro”, Bari, Italy; 7 Department of Biosciences, Biotechnology and Pharmacological Sciences, University Medical School “A. Moro”, Bari, Italy; 8 Department of Emergency Surgery and Organ Transplantation, Division of Veterinary Clinics and Animal Productions, University Medical School “A. Moro”, Bari, Italy; University of Catania, Italy

## Abstract

Failure of intestinal anastomosis is a major complication following abdominal surgery. Biological materials have been introduced as reinforcement of abdominal wall hernia in contaminated setting. An innovative application of biological patch is its use as reinforcement of gastrointestinal anastomosis. The aim of study was to verify whether the bovine pericardium patch improves the healing of anastomosis, when *in vivo* wrapping the suture line of pig intestinal anastomosis, avoiding leakage in the event of deliberately incomplete suture. Forty-three pigs were randomly divided: Group 1 (control, n = 14): hand-sewn ileo-ileal and colo-colic anastomosis; Group 2 (n = 14): standard anastomosis wrapped by pericardium bovine patch; Group 3 (n = 1) and 4 (n = 14): one suture was deliberately incomplete and also wrapped by patch in the last one. Intraoperative evaluation, histological, biochemical, tensiometric and electrophysiological studies of intestinal specimens were performed at 48 h, 7 and 90 days after. In groups 2 and 4, no leak, stenosis, abscess, peritonitis, mesh displacement or shrinkage were found and adhesion rate decreased compared to control. Biochemical studies showed mitochondrial function improvement in colic wrapped anastomosis. Tensiometric evaluations suggested that the patch preserves the colic contractility similar to the controls. Electrophysiological results demonstrated that the patch also improves the mucosal function restoring almost normal transport properties. Use of pericardium bovine patch as reinforcement of intestinal anastomosis is safe and effective, significantly improving the healing process. Data of prevention of acute peritonitis and leakage in cases of iatrogenic perforation of anastomoses, covered with patch, is unpublished.

## Introduction

Failure of gastrointestinal anastomosis results in dehiscence, leaks and fistulas, and is considered a major complication following abdominal surgery. Despite improved surgical technique, the reported incidence of gastrointestinal anastomosis leakage ranges from 2% to 10% [1]–[5], and is associated with both increased morbidity (20–30%) and mortality (7–12%) [1]–[5].

Hypoalbuminemia, chronic obstructive pulmonary disease, colon cancer and IBD have been identified as significant risk factors for anastomotic leakage [1], [3], [4], [6]. However, wide resection margins, absence of tension at the level of the suture site and resection along anatomic blood supply may decrease the risk [1], [6]. By contrast, hypovolemia, blood transfusions, besides surgical skill, prolonged operative time and difficult operative procedures, have been associated with anastomotic leakage development [1], [6].

Biological materials have been introduced in general surgery as reinforcement of abdominal wall hernia in contaminated setting, when the use of alloplastic meshes is contraindicated [7]. Hand to hand with the success of bovine pericardium for valves and patch grafts in cardiac surgery [8], [9], this biomaterial has been considered suitable in place of dura mater in anterior abdominal wall defects in pediatric surgery [10], [11] and then for the treatment of the patients affected by incisional hernia particularly in the contaminated or urgent context. An innovative application in this respect is the use of the biomaterials as reinforcement of the gastrointestinal anastomotic suture line [12]–[21]. In particularly, the available experimental data showed that the mechanical anastomoses buttressed with bovine pericardium [13], [14] or small intestinal submucosa had greater bursting strength as compared to non-buttressed anastomoses [15], [19] and a wound healing improvement [14], [17] has been demonstrated. Moreover, a colic perforation treated by positioning a resorbable bilayer collagen band of bovine origin in a pig model showed results very encouraging [21].

The aim of the study was to verify whether the bovine pericardium patch improves the healing of anastomosis, when affixed in vivo on the hand-sewn suture line of ileo-ileal and colo-colic anastomosis of the pigs. A further end-point was to see whether the patch is able to avoid the anastomotic leakage in the case of deliberately incomplete suture. For this, we used a pig model undergoing intraoperative and histological evaluation, and biochemical, tensiometric and electrophysiological measurements of intestinal specimens because these animals recapitulate several key features of human anatomy and physiology of the grastrointestinal tract.

## Materials and Methods

### Animals and Ethics Statement

After approval by the Italian Ministry of Health (protocol number: 02/2010) and in strict accordance with the recommendations in the Guide for the Care and Use of Laboratory Animals of the National Institutes of Health, between September 2010 and April 2012, forty-three domestic pigs (Landrace; female; mean age 5.3±2.2 months; weight 38.7±9.2 Kg) were sourced from commercial piggery affiliated with the Division of Veterinary Clinics and Animal Productions of University Medical School “A. Moro” of Bari (Italy), and were included in this study and underwent two surgical procedures. Vendor health reports indicated that the animals were free of known viral, bacterial and parasitic pathogens. The pigs were weighed before surgery and at regular interval during the experimental period. After a preoperative fasting time of 24 hours, all surgery was performed under an aseptic setting and general anesthesia, and all efforts were made to minimize suffering. At the end of the second surgical procedure, pigs were euthanized with a bolus of thiopental followed by a bolus of KCl. All pigs received ampicilline (25 mg/kg every 12 hours) and tramadol (5 mg/kg daily) for seven days after surgery. Pigs were fed, leaving water *ad libitum,* and were monitored daily in order to detect any alteration of the clinical conditions (food intake and weight loss; urine and feces production; rectal temperature and behavior changes).

### Study groups and Surgical Procedure I

**Figure 1:**
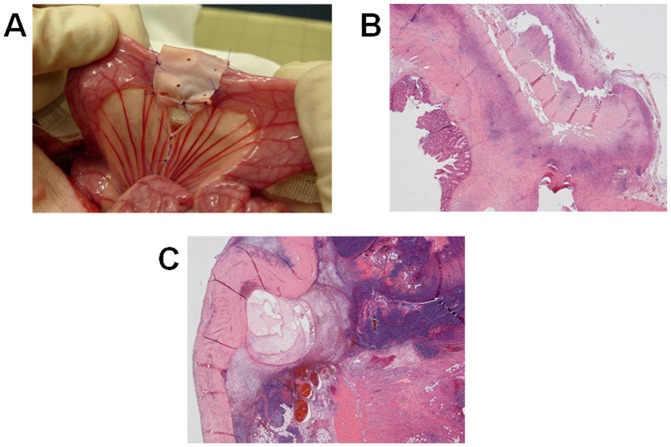
(A–C) Intraoperative image and histological findings of intestinal anastomosis wrapped by pericardium bovine patch. Ileo-ileal anastomosis wrapped by a 50×20 mm one layer pericardium bovine patch (A); at day 7 (B) the patch is enveloped by an heavy lympho-isthyocitic infiltrate without essudation on the serosal surface (Group 2) and after 48 h (C) the necrotic tissue is well contained into the intestinal wall by the patch (Group 4), with minimal leukocyte infiltration of the serosal surface (Haematoxylin-Eosin 200X original magnification).

The peritoneal cavity was entered *via* a midline incision and a segment of colon 30 cm from the anal verge and of ileum 30 cm from the cecum verge were resected. The animals were randomly assigned to four groups: 1) Group 1 (control, n = 14): the hand-sewn ileo-ileal and colo-colic anastomosis were performed using single layer of interrupted suture (PDS 3/0, Ethicon, Germany) according to *Gambee;* the distance between the single sutures were 3–4 mm; 2) Group 2 (n = 14): the standard anastomosis were wrapped by a 50×20 mm single layer pericardium bovine patch (Tutomesh®, Tutogen Medical GmbH, Germany); in order to allow some swelling during healing, 360° anastomotic sealing was achieved by a 5 mm over-lapping of the two ends of the patch at the mesenterial side of the anastomosis without fixing the ends to each other (Fig. 1A); 3) Group 3 (n = 1): one suture of the anastomosis was deliberately performed incomplete; 4) Group 4 (n = 14): one suture of the anastomosis was deliberately performed incomplete and wrapped by the pericardium bovine patch. The abdominal cavity was closed in layers with absorbable sutures in all pigs. Procedures were carried out by the same surgeon (M.T.) and the randomization was performed by using numbered and sealed envelopes that were opened at the beginning of the operation.

### Surgical Procedure II and Macroscopic examination

In Group 1, 2 and 4, a relaparotomy was performed 48 h (4 pigs for each group), 7 and 90 days after (5 pigs for each group); the animals of the Group 3 were reoperated 48 h thereafter only. The abdominal cavity was inspected for any kind of free fluid collection and anastomotic leakage, stenosis, abscess, peritonitis or intra-abdominal adhesions. Quantity and quality of adhesions were examined in a scoring system (0–3 points). In order to perform the microscopic, biochemical, tensiometric and electrophysiological studies, an intestinal tract was dissected four centimeters upstream and downstream the surgical site.

### Preparation of histological specimens and Microscopic examination

All the samples were fixed with neutral buffered formalin for 24–48 hours and paraffin- embedded; consecutive sections of 3 µm thickness were longitudinally cut to include the anastomosis and the adjacent intestinal tracts, in order to observe the whole section from the mucosa to the serosa, and were stained with haematoxylin-eosin and periodic acid-Schiff stains.

Evaluation was carried out by the same pathologist blinded for the experimental protocol. For each section the following histological parameters were evaluated: grade of inflammation, parietal fibrosis, integrity of the mucosal layer, serosal status, and the grade of granulocyte infiltration of the membrane in cases with the biological mesh. Phlogosis, fibrosis and mesh infiltration were employed for scoring system (0–4 points).

### Biochemical studies

Immediately after surgery, the tissue of representative segments of ileo-ileal and colo-colic anastomosis (Group 1, 2) was disrupted by a glass-Teflon homogenizer and mitochondria were isolated according to *Bookelman et al*. [22]. The mitochondrial respiration was measured polarographically with a Clark-type oxygen electrode in a water-jacketed chamber, magnetically stirred at 37°C. Respiration rates were expressed as nanomoles of molecular oxygen consumption *per* minute *per* milligram of mitochondrial protein [23], after addition of substrates and inhibitors of oxidative phosphorylation. To measure the rotenone sensitive reduced nicotinamide adenine dinucleotide (NADH)-ubiquinone oxidoreductase activity, mitochondria were exposed to ultrasound energy for 15 s at 0°C [23]. Cytochrome c oxidase activity was determined on mitochondria [23], following the oxidation of ferrocytochrome c 10 μM. Citrate synthase activity [23] was used as mitochondrial matrix enzymatic marker. For detection of reactive oxygen species (ROS) in ileo-ileal and colo-colic anastomosis whole tissue homogenate, fluorimetric analysis was used [23] with a Jasco FP6200 spectrofluorimeter. All activities were expressed as nanomoles of substrates *per* minute *per* milligram of mitochondrial protein.

### Tensiometric studies

Tensiometric studies of freshly excised colic and ileal smooth muscle specimens (Group 1, 2) were conducted at 48 hours, 7 and 90 days after surgery. Specimens were mounted in an organ bath filled with modified Krebs' solution (20 ml at 37°C gassed with O2/CO2 (95%/5%), pH 7.4). Isometric tension was measured with a strain gauge transducer (cat. 7003 Basile, Milan, Italy) connected to data acquisition system (PowerLab and Chart 4.1.2 ADInstruments, Castle Hill, Australia). After 45 minutes equilibration, an initial load of 1.0 g tension was applied to the tissue. Contractile responses were measured to acetylcholine (ACh, 10^−7^–10^−4^ M) and KCl (80 mM).

### Electrophysiological measurements

Transepithelial potentials (V_T_), resistance (R_T_) and short circuit current (I_SC_) were measured as markers of colic and ileal mucosa transport efficiency at baseline, early stage (days 2 and 7, tissues pool), and late stage (day 90) after surgery in Groups 1, 2. Freshly excised mucosa was placed in a cooled modified Krebs' bicarbonate/phosphate buffer solution (at 37°C gassed with O2/CO2 (95%/5%), pH 7.4) and mounted in a Ussing chambers (Mussler Scientific Inst., Aachen, Germany) with an exposed area of 1 cm^2^. Two pairs of Ag/AgCl electrodes were used to monitor V_T_ (mV) and R_T_ (Ω.cm2). I_SC_ (μA/cm^2^) was measured with the V_T_ clamped to 0 at 5 min intervals. At fixed intervals of 1 min a transepithelial bipolar current pulse (I) of 1 µA amplitude and 200 msec duration was applied to tissue and R_T_ calculated from the change in open-circuit voltage (ΔV_T_) according to Ohm's law (R_T_ = ΔV_T_/I). Experiments were conducted simultaneously on multiple specimens, each from an individual pig. Electrical parameters were measured by the software Clamp (v. 2.14, Aachen, Germany) and recorded for 60 min after an initial equilibration time of about 30 min.

### Statistical analysis

Data are given as means ± SEMs. Infra-group comparisons were made using median test with the two sided p-value computed using Fisher's exact test. For biochemical studies, one-way or two-way analysis of variance, as appropriate, followed by paired Student *t*-test or Newman-Keuls multiple comparison tests were used. For electrophysiological studies, data of the measurements during 60 min were compared between control and treated tissues were assessed using the Student *t*-test for unpaired data between the data average of early and late follow-up days *vs* day 0 and for anastomosis with patch *vs* anastomosis without patch. Data were analyzed by Stata 12 software (StataCorp LP, College Station, Tex, USA). *P* values of <0.05 were considered statistically significant.

## Results

### Macroscopic examination

Tables 1 and 2 summarize the intraoperative findings at relaparotomy. In the control group, one leakage was revealed in a colo-colic anastomosis 48 h after surgery. No stenosis, abscess or peritonitis was found in this group and adhesions were progressively observed in the subgroups. In Group 2, the intraoperative evaluation of the abdominal cavity lacked anastomotic leaks, intraabdominal abscess, stenosis or peritonitis. Moreover, adhesion rates (quantity and quality; Table 2) were significantly less than the control group (p = 0.006 and p = 0.018, respectively). As expected, in Group 3, peritonitis was evident and adhesions were extremely solid. By contrast, in Group 4, no leaks, stenosis, abscess and peritonitis were seen in any of the pigs and adhesion rates progressively increased, but less than Group 1 again (p = 0.016 and  = 0.006, respectively). No shrinkage and displacement of prosthesis was seen either in Group 2 or 4.

**Table 1:** Intraoperative results.

Group	Relaparotomy	Leak	Stenosis	Abscess	Peritonitis	Pericardium bovine patch	
						Shrinkage	Displacement
	48h (4)	1*	0	0	0	-	-
1 (n)	7 pod (5)	0	0	0	0	-	-
	90 pod (5)	0	0	0	0	-	-
	48h (4)	0	0	0	0	0	0
2 (n)	7pod (5)	0	0	0	0	0	0
	90 pod (5)	0	0	0	0	0	0
3 (n)	48h (1)	1**	0	0	1	-	-
	48h (4)	0	0	0	0	0	0
4 (n)	7pod (5)	0	0	0	0	0	0
	90 pod (5)	0	0	0	0	0	0

h: hours; pod: post-operative day; *colo-colic anastomosis; **both anastomosis.

**Table 2:** Mean Adhesion Score.

Adhesion rate I quantity*	Group				*P*	
	1	2	3	4	2 *vs* 1	4 *vs* 1
48h	1±0	0±0	3	0.3±0.3	0.029	NA
7 pod	2.4±0.2	0.2±0.2	-	1.0±0.0	0.008	0.008
90 pod	2.8±0.2	1.4±0.2	-	2.0±0.0	0.048	0.048
*Total*	2.1±0.2	0.6±0.2	-	1.1±0.2	0.006	0.016
Adhesion rate II quality**						
48h	1±0	0±0	3	0.5±0.3	0.029	NA
7 pod	2.2±0.4	0.2±0.2	-	1.6±0.2	0.048	0.444
90 pod	3±0	1.4±0.2	-	2±0	0.008	0.008
*Total*	2.1±0.3	0.6±0.2	-	1.4±0.2	0.018	0.006

h: hours; pod: post-operative day. Mean score ± SEM. *Adhesion rate I: 0 =  no adhesion; 1 =  adhesions with one structure; 2 =  adhesions with two structures; 3 =  adhesions with three or more structures. **Adhesion rate II: 0 =  no adhesions; 1 =  light adhesions; 2 =  fixed adhesions; 3 =  solid adhesions, only removable with damage.

### Histological examination

Table 3 shows the microscopic findings of the anastomosis at the relaparotomy. In control group, a heavy inflammatory infiltrate was observed at early stage, decreasing progressively and being virtually absent on day 90 postoperatively. Conversely, the fibrogenic process was initially evident from the 7^th^ postoperative day and completed on the 90^th^. The mucosal surface and the serosal layer rapidly recovered. In Groups 2 (Fig. 1B) and 4 (Fig. 1C), there was no statistical difference in comparison with the control group in terms of the phlogosis. Moreover, the fibrotic reaction was statistically less evident in Groups 2 and 4 (both p = 0.033) compared to the control. Indeed, a significant granulation tissue was initially present at the border of the bovine pericardium patch and subsequently in the deeper areas. On the 90^th^ day there were only small, hardly recognizable fragments of the patch surrounded by macrophages and lymphocytes. While the mucosal surface rapidly recovered, the serosa became smooth only 90 days after the operation. In the Group 3, the inflammatory cell infiltrate and the fibrin deposition were massive and diffuse.

**Table 3:** Microscopic Findings and Mean Histological Score.

Phlogosis*	Group				*P*	
	1	2	3	4	2 *vs* 1	4 *vs* 1
48h	4±0	3.8±0.3	4	4±0	NA	NA
7 pod	3.4±0.2	3.4±0.2	-	3.4±0.2	1.000	1.000
90 pod	0.2±0.2	0.4±0.2	-	0.4±0.2	1.000	1.000
*Total*	2.4±0.5	2.4±0.2	-	2.5±0.5	1.000	1.000
Fibrosis*						
48h	0.5±0.3	0.3±0.3	4	0.3±0.3	1.000	1.000
7 pod	2.2±0.4	1.8±0.2	-	1.2±0.2	0.444	0.206
90 pod	3.8±0.2	2.2±0.2	-	2.2±0.2	0.048	0.048
*Total*	2.3±0.4	1.5±0.2	-	1.3±0.2	0.033	0.033
*Mucosa*						
48h	ulcerated	ulcerated	ulcerated	ulcerated	-	-
7pod	normal	eroded	-	eroded	-	-
90 pod	normal	normal	-	normal	-	-
*Serosa*						
48h	thickened	normal	thickened	normal	-	-
7pod	normal	thickened	-	thickened	-	-
90 pod	normal	normal	-	normal	-	-
Mesh infiltration*						
48h	-	0.3±0.3	-	0.3±0.3	-	-
7pod	-	1.4±0.2	-	1.4±0.2	-	-
90 pod	-	0.2±0.2	-	0.2±0.2	-	-
*Total*	-	0.6±0.2	-	0.6±0.2	-	-

h: hours; pod: post-operative day; mean score ± SEM. *Score 0-4: 0 =  absent; 1 =  minimal; 2 =  moderate; 3 =  distinctive; 4 =  severe.

### Biochemical studies

**Figure 2:**
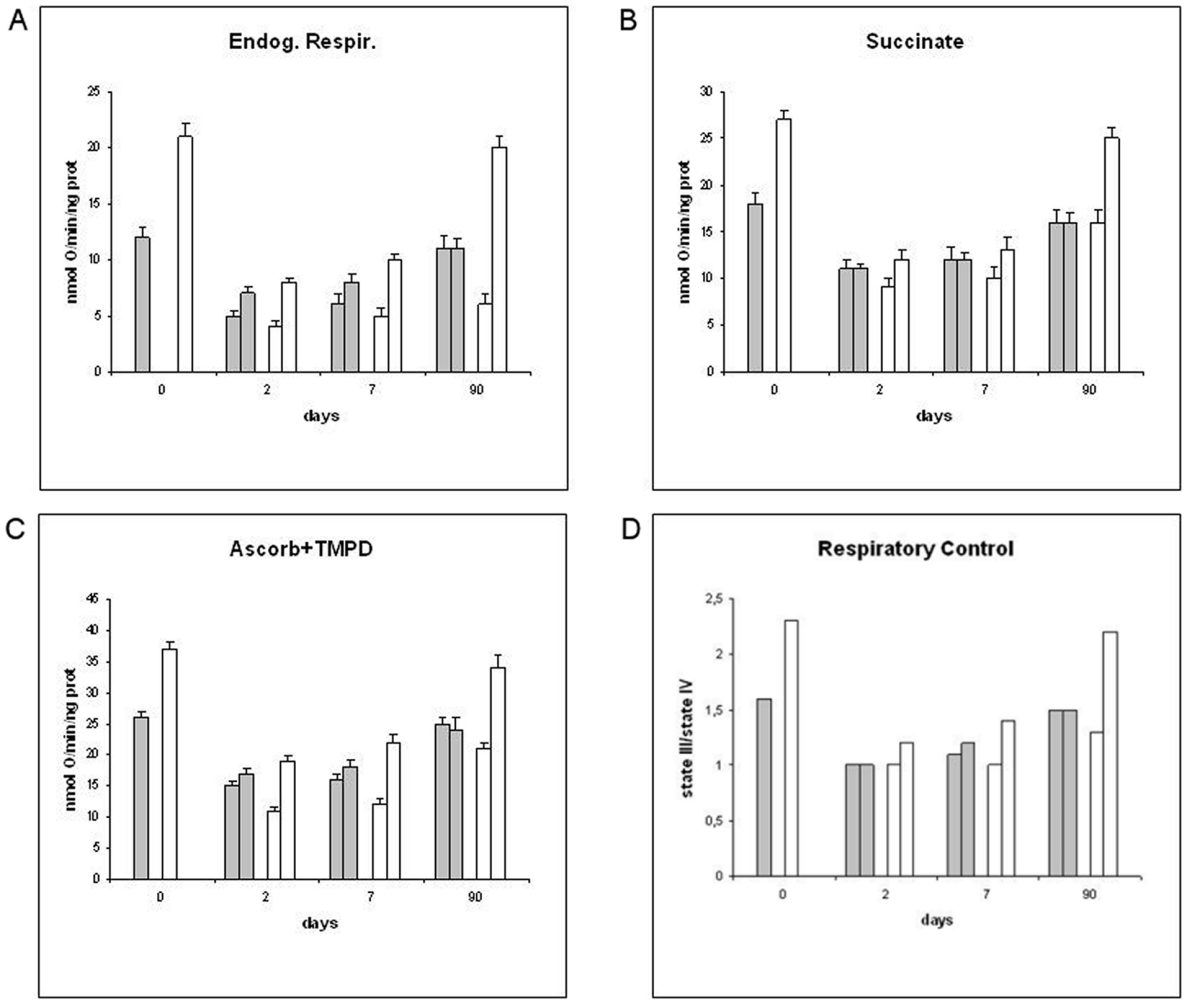
(A–D) Mitochondrial respiration. Mitochondrial respiratory activities, from endogenous substrates (A), succinate (B) and ascorbate (C), and mitochondrial respiratory control ratios from endogenous respiration (D) in ileo-ileal (gray bars) and colo-colic (white bars) anastomosis. In each coupled bars values from Group 1 (left) and Group 2 (right) are compared.

**Figure 3:**
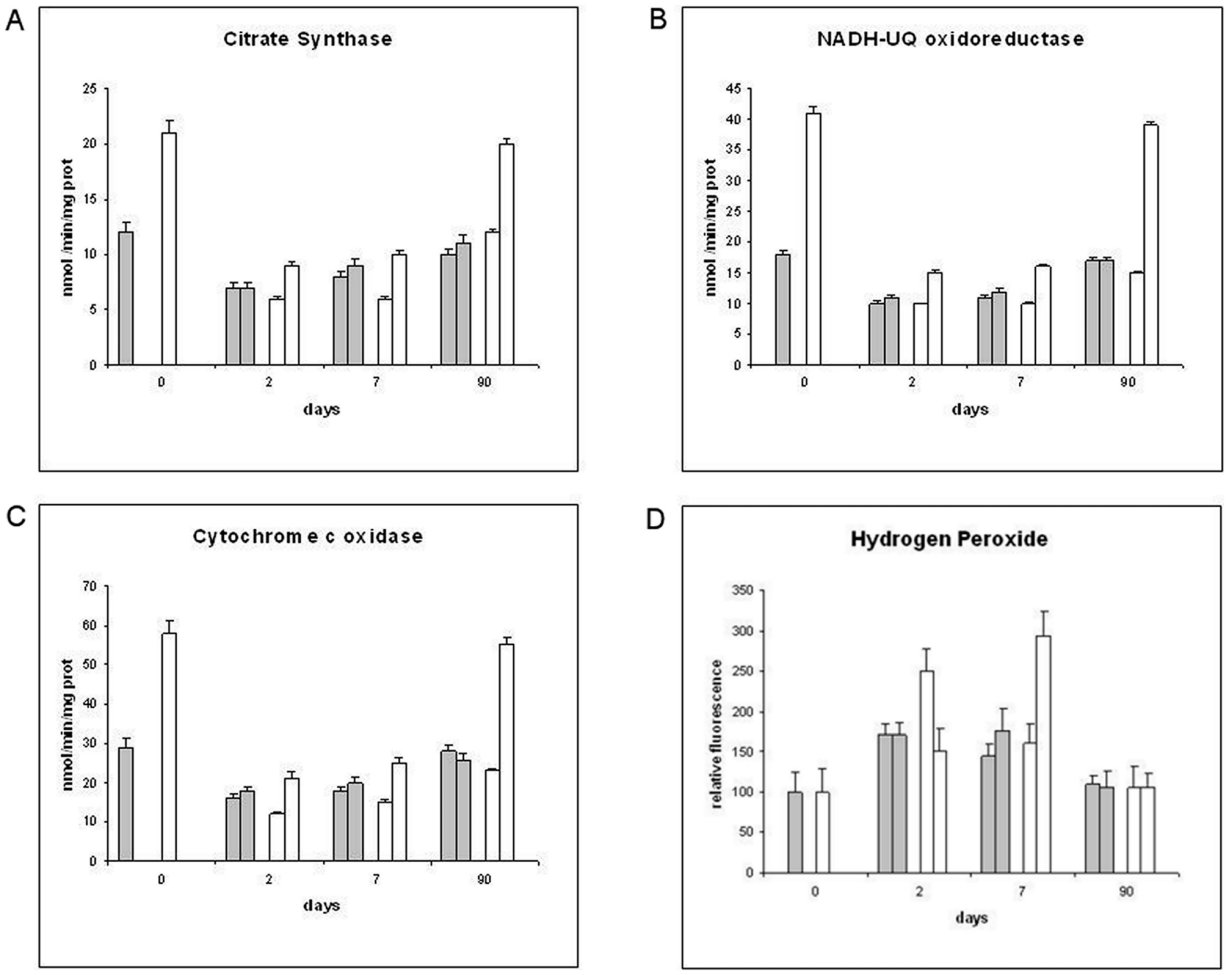
(A–D) Analysis of the others mitochondrial activities and reactive oxygen species. Mitochondrial activities of citrate synthase (A), NADH-ubiquinone oxidoreductase (B), cytochrome c oxidase (C) and hydrogen peroxide production (D) in ileo-ileal (gray bars) and colo-colic (white bars) anastomosis. In each coupled bars values from Group 1 (left) and Group 2 (right) are compared.

We measured the mitochondrial respiratory activity by endogenous substrates (NADH-dependent respiration through complex I, III and IV; Fig. 2A) and from added substrates, like succinate (respiration through complex II, III and IV; Fig. 2B) and ascorbate (final respiratory step through complex IV; Fig. 2C), in ileo-ileal and colo-colic anastomosis samples (Group 1, 2). In Fig. 2D, respiratory control ratios indicates the coupling of mitochondria, oxidative phosphorylation efficiency and adenosine triphosphate (ATP) production. In the same samples, at each time point, the enzyme marker for mitochondrial matrix (citrate synthase; Fig. 3A), and the enzymatic activities of complex I (NADH-ubiquinone oxidoreductase; Fig. 3B) and IV (cytochrome c oxidase; Fig. 3C) were measured. Within 48 hours after anastomosis, mitochondrial respiration (Fig. 2A–C) and enzymatic activities (Fig. 3A–C) showed a marked decline of all functional parameters in ileo-ileal and colo-colic anastomosis, which lasted for one week. After this phase, the recovery showed a difference between the two intestinal parts. Specifically, the biochemical parameters of ileo-ileal anastomosis showed a gradual recovery and in three months were almost fully restored to the initial values, without a significant influence by the patch (Group 2). On the contrary, the parameters of colo-colic anastomosis showed no recovery even after three months, except when the patch was applied, with a partial early recovery after two days, which was full after three months. Uncoupling of mitochondria from two days to one week after anastomosis was observed in both colo-colic and ileo-ileal anastomosis, with the latter being more affected (Fig. 2D). The Tutomesh®, once again, was shown to improve the recovery of mitochondrial coupling in colo-colic anastomosis from one week to three months. Measurement of hydrogen peroxide generation in whole tissue homogenates after anastomosis showed significant differences between ileo-ileal and colo-colic anastomosis (Fig 3D). In particular, in ileo-ileal anastomosis a significant increase of H_2_O_2_ production was detected from two days to one week after anastomosis, before returning to basal levels after three months and this did not seem to be significantly influenced by the patch. In classic colo-colic anastomosis, however, the increase of H_2_O_2_ generation was significantly higher two days after anastomosis and decreased progressively in the subsequent days reaching normal basal values after three months. Unlike this application of Tutomesh®, in colo-colic anastomosis it appeared to limit the early increase of H_2_O_2_ production, two days after anastomosis, which was delayed to a threefold increase at one week and gradually decreased to normal basal values in three months.

### Tensiometric studies

**Figure 4:**
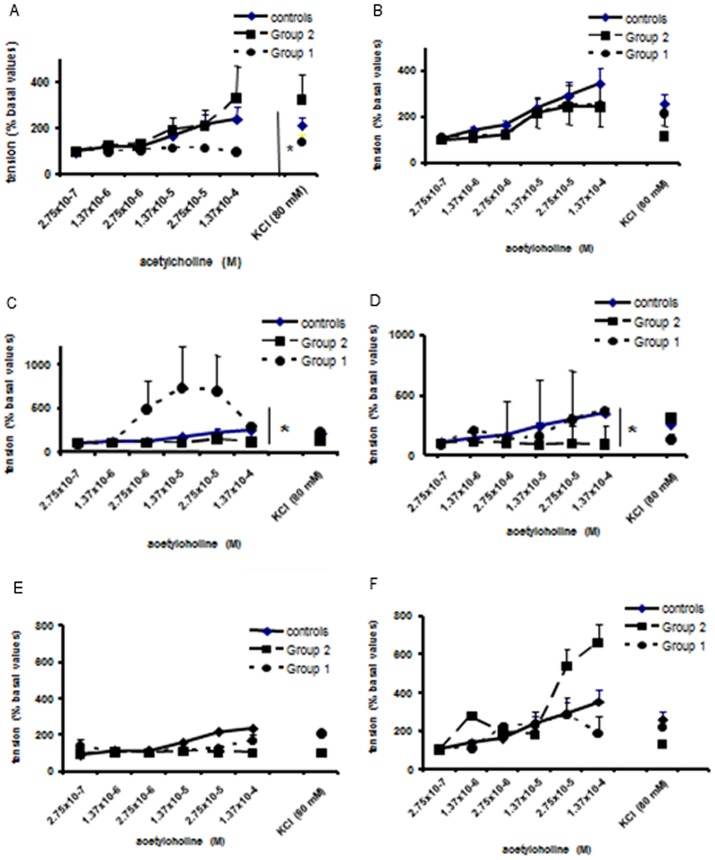
(A–F) Tensiometric studies. Colic (A,C,E) and ileal (B,D,F) contractility in Group 1 and 2 at 48 hours, 7 and 90 days after surgery, respectively.

At baseline, ACh induced dose-response contractions in ileum and colon specimens (controls), which were slightly more pronounced in ileum. The response to KCl was comparable in both bowel tracts. Forty-eight hours after surgery (Fig. 4A), the contractile response of the colon to ACh was lower (p<0.05) in Group 1 *vs* control, while Tutomesh® prevented this effect. No difference was found in ileal specimens (Fig. 4B). Seven days after surgery the contractile response of the colon to ACh increased in Group 1 (p<0.05) *vs* control, but was unchanged in Group 2 (Fig. 4C). In the ileum, Tutomesh® determined a reduced contractile response to ACh (p<0.05), while Group 1 response was similar to control (Fig. 4D). Ninety days after surgery, the response to ACh or KCl was comparable in different surgical treatments both in the colon and ileum (Fig. 4E,F). The response to KCl was similar in colic and ileal specimens (Fig. 4A–F).

### Electrophysiological measurements

**Figure 5:**
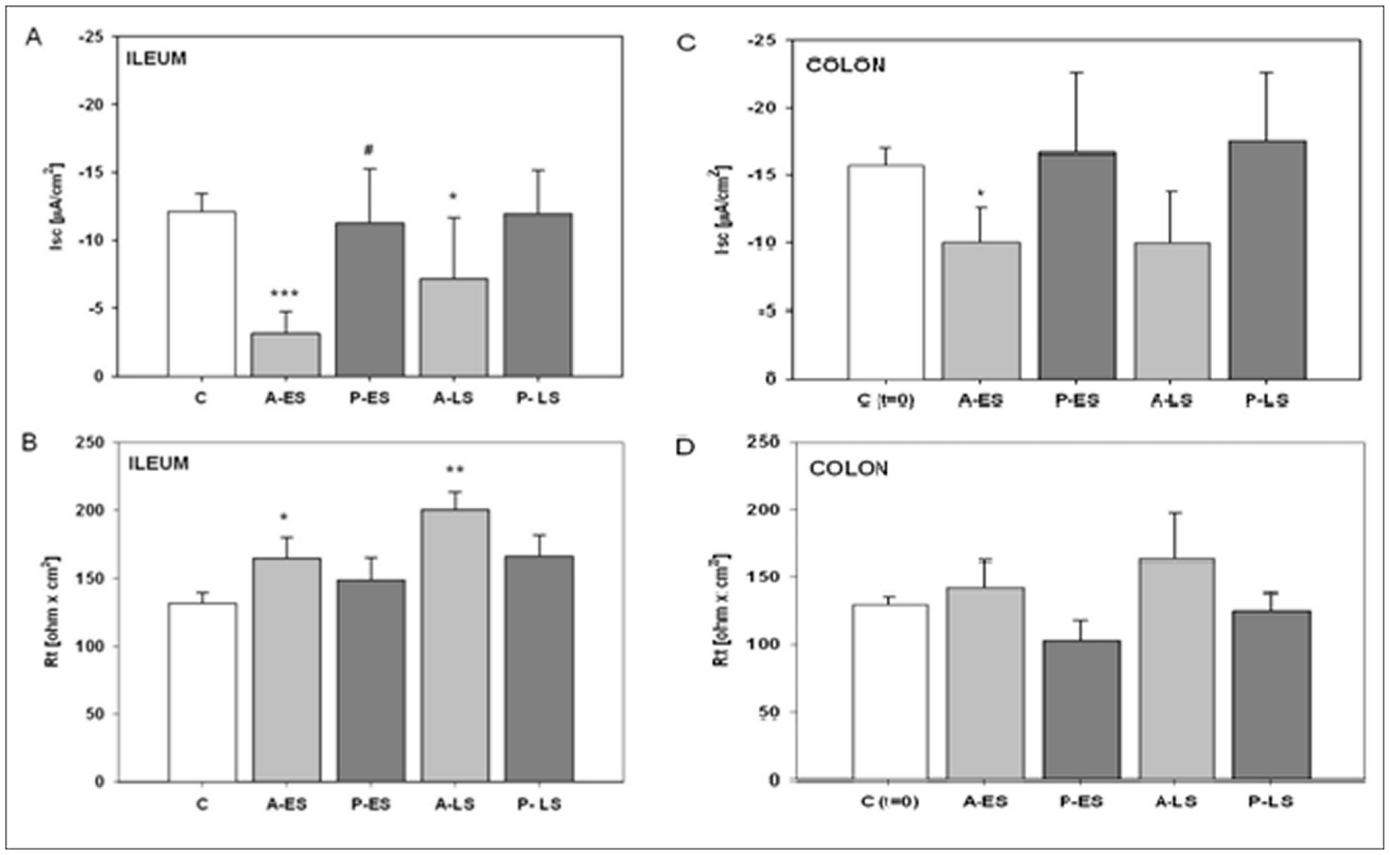
(A–D) Electrophysiological measurements. Short circuit current (I_SC_) and tissue resistance (R_T_) in ileal and colic specimens. Bars represent mean ± SEM in C (before surgery, N = 15), A-ES (after surgery without patch, early stage, N = 15), P-ES (after surgery with patch, early stage, N = 15), A-LS (after surgery without patch, late stage, N = 10), P-LS (after surgery with patch, late stage, N = 10) in ileal and colic specimens, respectively. Significance was determined by Student's t test for unpaired data of follow-up stage *vs* control (*p≤0.05; **p≤ 0.002; ***p≤0.0001) and for anastomoses with patch *vs* anastomoses without patch control (#: p≤0.05).

At baseline, the ileum showed I_SC_ −12.1±1.3 μA/cm^2^, R_T_ 131.2±8.1 Ω. cm^2^, and V_T_ 1.1±0.2 mV, lumen negative (N = 15). The colon showed I_SC_ −14.7±1.2 μA/cm^2^, R_T_ 126.6±6.9 Ω. cm^2^, and V_T_ 0.59±0.1 mV, lumen negative (N = 15). In the ileo-ileal anastomosis the early stage, was associated with significantly decreased I_SC_ (−74.1%, p<0.0001 *vs* control) and increased R_T_ (+25.7%, p<0.05 *vs* control) (Fig. 5A,B). A similar trend (i.e. −35%) existed for V_T_ (data not shown). In the late stage, I_SC_ remained below the control value (−40.9%, p<0.05 *vs* control; Fig. 5A), R_T_ increased significantly up to 53% (p<0.02 *vs* control; Fig. 5B), and V_T_ decreased by 57.5% (p<0.02 *vs* control). The presence of colo-colic anastomosis in the early stage was associated with significantly decreased I_SC_ (−36,4%, p<0.05 *vs* control; Fig. 5C), a tendency of increased R_T_ (Fig. 5D) and reduced V_T_. In the late stage, I_SC_ remained stable, while R_T_ and V_T_ tended to increase compared to the control. Affixing a pericardium bovine patch on ileal anastomosis prevented the electrophysiological changes seen with anastomosis alone (I_SC_ virtually unchanged in the early and late stages, Fig. 5A) while R_T_ (Fig. 5B) and V_T_ did not increase. In the colon, likewise, the prosthesis prevented the changes of I_SC_, R_T_ and V_T_ (Fig. 5C–D).

## Discussion

Our experimental study is the first in literature to investigate whether a pericardium bovine patch, wrapping ileo-ileal and colo-colic hand-sewn anastomosis in pigs, seals the suture line and promotes processes of anastomotic healing. Dehiscence of anastomosis is a disturbing, common and severe complication after bowel resection, with an incidence ranging from 0.5 to 30% after large bowel resection and to about 6% after small bowel resection [1]–[5].

Several studies have demonstrated that the early integrity of the anastomosis depends not only on the correct surgical performance, but also on the suture-holding property of the sub-mucosal layer and on formation of a fibrin seal on the serosa [24]. Furthermore, the process of anastomotic healing is related to the structure and arrangement of the collagen matrix [24].

Despite the fact that many risk factors for postoperative leakage have been analyzed in literature [1], [3], [4], [6], few clinical and experimental studies have focused on the best prevention techniques of anastomotic dehiscence [25], [26]. However, operative and post-operative treatments, allowing suitable oxygenation and blood perfusion of the anastomotic side and stimulating the angiogenesis, with infusion of grow factors or inhibitors of metalloproteinase, have been investigated [25], [26]. Some studies investigated whether the reinforcement of anastomosis with biological or synthetic materials was able to prevent the anastomotic leakage. Reports regarding the use of synthetic materials are, however, uncommon and with discordant outcomes [27], [28]. Hand in hand with the use of the biologic mesh in abdominal wall repair, many experimental studies on their application as reinforcement of anastomosis have spread, and the bovine pericardium [13]–[16], small intestinal submucosa [17]–[19] and porcine dermis [20] are the biomaterials used. The biological materials are all basically composed of an extracellular matrix deprived of its cellular components. The extracellular matrix serves as scaffold for the remodeling process by host through the connective tissue ingrowth and cellular colonization and proliferation. Reports regarding the use of the bovine pericardium are experimental [13]–[15] or clinical studies [16] and the anastomoses have been performed using a circular stapler with the introduction of bovine pericardium as a buttressing material to reinforce staple lines.

In this study, the anastomosis was performed using a single layer of interrupted suture according to *Gambee.* In accordance with knowledge of the healing phases of anastomosis, the surgical procedure II was carried out in the early and late phase (at 48 h, 7, and 90 days, respectively after the first operation). However, the previous experimental studies analyzed the early effect (after a few hours) of the pericardium bovine buttress on the anastomosis [13], [15] and only *Hagerman et al* [14] performed the evaluation in late phase of suture healing. In this study, we provide a complete overview of key events involved in anastomosis healing with and without bovine patch. By using highly integrated and translational methodologies, we describe a detailed intraoperative evaluation, histological and biochemical analyses, and tensiometric and electrophysiological studies on intestinal specimens after the surgical procedure II for each group of pigs. Overall, the whole intestinal wall from mucosa to muscle and serosa was fully assessed by morphological and functional methods. Macroscopic side effects of reinforcement with pericardium bovine patch were investigated in intraoperative setting II. In accordance with the uneventful convalescence of the pigs in the groups with the wrap of pericardium bovine on anastomosis, no leak or stenosis or abscess and peritonitis were found in early and late relaparotomy, nor were any cases of displacement and shrinkage of the mesh. Some Authors described that the reinforcement with collagen fleeces could cause intestinal obstruction, resulting from the phenomena of shrinkage and displacement of the prosthesis [12], [17]. Moreover, we found decreased rate of intra-abdominal adhesions compared to standard anastomoses, while other studies showed a similar entity of adhesions [17], [18]. As expected, when the iatrogenic perforation had been performed without any reinforcement, a diffuse and severe peritonitis was the intraoperative finding.

On the other hand, an original finding is that the absence of leak, stenosis, abscess and peritonitis was also found when one suture of the anastomosis was performed deliberately incomplete and wrapped by the pericardium bovine patch. Moreover, intra-abdominal adhesions in this group were less significant in comparison with the control again. The same biological features, for which the bovine pericardium has been employed widely for the abdominal wall hernia repair, could explain this important data. The bovine pericardium is an acellular membrane of pure collagen with native structure, not cross-linked, made up of multidirectional fibers with adequate tensile strength and representing the scaffold for replacement by new endogenous tissue. The remodeling process of the mesh by host determines complete degradation by fibroblasts with deposition of autologous tissue, without foreign body reaction, or fibrous capsule formation, or shrinking, or adhesion formation and fast peritoneal regeneration. We hypothesized that the biological wrap has obstructed the leak through the small iatrogenic perforation, firstly with mechanical action and definitively with the improvement of healing of suture. The mechanism of healing could be the result between the combination of immediate action as cap on the anastomotic dehiscence, and the known remodeling process of the mesh, through the gradual degradation of bovine pericardium *via collagenase*, and the rapid cellular ingrowth. Obviously, this effect is possible when a small leakage and incomplete anastomotic dehiscence occurs. Confirming the buffering effect of the pericardium bovine patch in order to avoid the anastomotic leakage in the cases of iatrogenic perforation and the improvement on the healing of the tissue, the microscopic findings included only a moderate granulocyte infiltration limited to the patch, but no signs of peritonitis. Statistically, the fibrosis was less induced than the control and on 90^th^ day, the remodeling process of the mesh was complete. As reported previously [17], our histological examination showed a migration of fibroblasts into the pericardium bovine patch, a regeneration of the bowel layer at the anastomotic site, with the improvement of healing of the host tissue. This improvement of healing was shown first in the site of iatrogenic perforation.

There is currently a lack of biochemical, tensiometric and electrophysiological studies regarding the effect of the pericardium bovine patch on the intestinal anastomosis in literature. The biochemical results indicated that the colon relies differently on mitochondrial aerobic metabolism than the ileum, according to respiratory and enzymatic activities of the basal samples, which are about twofold higher in colon, as found from previous observations by our group [29], [30]. Confirming these previous experimental results [29], [30], after surgical treatment, a temporarily ischemic condition, associated to some necrosis and release of inflammatory mediators, may generate a local anaerobic environment and consequently a switch to glycolytic metabolism for cellular ATP supply. Tissues depending on oxidative aerobic metabolism, like the colon, may suffer under these conditions with a lack of adaptation to glycolytic metabolism, whilst anaerobic metabolism can be better tolerated by the small bowel [29], [30]. This may explain the marked depression of mitochondrial functions in the colo-colic anastomosis, whilst the ileum can tolerate the post-anastomosis stress. Consequently, repair processes after anastomosis may be more efficient in the ileum that is easily able to proceed from the initial critical phase to the healing process, as shown by the recovery of mitochondrial parameters in the subsequent days, which are fully restored in three months. It appears that the colon does not comply well with the post-anastomosis stress, due to the cellular component of the tissue exhibiting high metabolic demand from oxidative metabolism, causing an irreversible loss of noble elements, which will not be replaced in the subsequent phases of healing. The loss of mitochondrial functions, indeed, does not seem to be restored to the initial values, even after three months.

The capacity of the ileum to tolerate the post-anastomosis stress may explain why the introduction of a patch support in the healing does not improve the whole process, as shown by the compared values of mitochondrial respiration and enzymatic activities. On the contrary, the affixing of the patch on the colic anastomosis produces a positive effect in the healing, as indicated by the data of mitochondrial functions, which perform better when compared to untreated samples and show an almost complete recovery in 3 months.

We have experimental evidence that the patch may play a role on the oxidative stress generated during the healing process of anastomosis, as shown by measurements of H_2_O_2_ production. In the ileo-ileal anastomosis, independently from the patch, a significant increase of ROS levels is observed from two to seven days after anastomosis, indicating that an inflammatory response and tissue regeneration take place contemporarily and obviously both are needed for the healing process (as also indicated by the presence of a heavy inflammatory infiltrate in the histological samples). In colo-colic anastomosis, without patch, the ROS levels have an early twofold increase at 48 hours, which declines in one week. This precocious oxidative stress could be an additional factor involved in the tissue damage that leads to mitochondrial dysfunction and lack of restoration of tissue function. On the contrary, when the patch is applied to the colo-colic anastomosis, the maximum increase of ROS is delayed at one week where it reaches a threefold level compared to basal production. After this peak, the ROS generation decreases and after one month their levels are normalized. The delay of oxidative stress in patch anastomosis could prevent damage to noble cells in the large bowel, like tissue stem cells, in the early stage of the repair processes, allowing a complete restoration of tissue functions and a decrease of fibrotic reaction in the subsequent stages. The protective effect of a patch is compatible with the histological observation of a moderate inflammatory infiltrate and the late increase of ROS can correlate with the appearance of a significant granulation tissue, which, at this final stage, is no more harmful for the repair process.

Regarding tensiometric evaluations, our results suggest that the use of the patch can preserve smooth muscle response to acetylcholine similar to the response of controls (samples without anastomosis) in colic specimens in the early postoperative time (48 h-7 days), while the colic preparations with traditional anastomosis showed contractility alterations compared to control. However, the use of pericardium bovine patch seems to impair the ileal contractile response at seven days after surgery. In late stages, the responses to acetylcholine were comparable to the specimens without anastomosis for both colon and ileum in which it is affixed to the mesh. It is conceivable that the decreased fibrotic process seen with the affixing of Tutomesh® might have preserved the smooth muscle function.

The results of electrophysiological parameters are able to describe fine changes occurring at the intestinal mucosal side, a very delicate monolayer structure that is highly sensitive to local and surrounding changes. Results are in good agreement with those reported for the pig by others [31]–[33], when also including tissue-specific differences between ileum and colon. Overall, our results suggest that subtle but important changes are observed in both ileum and colon in the cases of unprotected anastomosis. A number of factors, including early inflammatory changes, oxidative stress and late cicatricial tissue formation around the anastomosis, might impair both permeability and transport properties in the mucosa. In the ileum, the presence of the pericardium bovine patch clearly prevents the alterations following the traumatic effect of surgery. The colon appears to behave slightly differently, in that the transport properties are significantly reduced only at an early stage, while the permeability is less affected. Tutomesh®, however, appears to modulate and counteract the traumatic effect of surgery. Overall, our results suggest that application of the patch also improves the intestinal mucosal function, restoring almost normal transport properties. This is a process in line with the histological finding of less fibrotic reaction within the intestinal wall upon affixing of Tutomesh®.

## Conclusions

Our study demonstrated that the use of the pericardium bovine patch as reinforcement of the intestinal anastomosis is safe and effective. All performed analyses showed a significant improvement of the healing process of the anastomosis. Moreover, the histopathological data of the prevention of leakage into the abdominal cavity in cases of iatrogenic perforation of the ileal and colic anastomosis, covered with the wrap of bovine pericardium, resulting in prevention of acute peritonitis, is unpublished and surprising. The biochemical analysis confirmed that the post-anastomosis cellular stress, which probably involves inflammatory events, cytokines release, cell growth induction and anaerobic conditions, is better tolerated by the ileum, whilst the aerobic colon may suffer to a greater extent undergoing oxidative stress and mitochondrial failure, which are prevented by the use of a patch. The use of patch is advisable also for improving the healing process inasmuch as it is able to guarantee the efficient recovery of the functional transport properties of the intestinal epithelia and of the contractility. We suggest that our experimental results could be the basis for the multicenter controlled clinical trials in humans, comparing the outcomes of intestinal anastomosis performed with and without the bovine pericardium patch as reinforcement. The aim of these studies could be also to analyze the impact in terms of cost-benefit health, verifying a possible decrease of morbidity and mortality related to the healing of the anastomosis.
